# Research on Bearing Fault Diagnosis Based on GMNR and ResNet-CABA-MAGRU

**DOI:** 10.3390/s25113458

**Published:** 2025-05-30

**Authors:** Longfa Chen, Na Meng, Wenzheng Sun, Sen Yang, Shuo Tian, Yuguo Li

**Affiliations:** School of Intelligent Equipment, Shandong University of Science and Technology, Tai’an 271019, China; 202383230046@sdust.edu.cn (L.C.); 202383230038@sdust.edu.cn (W.S.); 202283230059@sdust.edu.cn (S.Y.); 202483230076@sdust.edu.cn (S.T.); 202483230068@sdust.edu.cn (Y.L.)

**Keywords:** deep learning, fault diagnosis, attention mechanism, Gram denoising module (GMNR)

## Abstract

Focusing on the problem that it is difficult to maintain a high diagnostic accuracy rate, short running time, and robust generalization capability in the face of a strong-noise environment in rolling bearing fault diagnosis, a bearing fault diagnosis model (GMNR-CABA-MAGRU) founded upon a new attention-mechanism-improved residual network (ResNet-CABA) and a Gram denoising module (GMNR) is proposed, and the CWRU bearing dataset is used for verification. Under the 0-load condition in a noise-free environment, the diagnostic accuracy of this model reached 99.66%, and the running time was only 52.74 s. Then, a bearing dataset with added Gaussian noise from −4 db to 4 db was verified, and this model was still able to maintain a diagnostic accuracy of 90.32% under the strong-noise environment of −4 db SNR. And migration experiments were carried out under different load conditions, and this model was also able to maintain a very high accuracy rate. Moreover, in all the above experiments, this model performed better than various comparative models. The developed framework demonstrated superior diagnostic precision, enhanced robustness, and improved generalization capability.

## 1. Introduction

In the field of modern mechanical equipment, the key role of rotating machinery cannot be ignored. However, bearing faults often occur in rotating machinery. Faults inside rotating machinery may destroy the accuracy of the equipment, and in extreme cases, due to the domino effect, catastrophic system-wide failures will be triggered. When a failure occurs, it can directly impact industrial processes, sometimes resulting in substantial financial losses or even harm to personnel. This highlights the importance of creating a dependable and rapid bearing fault diagnosis system to safeguard industrial machinery’s performance and operational safety.

The method of bearing fault diagnosis mainly includes two parts: extracting fault features and identifying and classifying fault states. In traditional bearing fault diagnosis methods, the extraction of bearing faults is mainly composed of signal processing methods and feature values. Signal processing methods include wavelet transform [1], empirical mode decomposition (EMD) [2], and variational mode decomposition (VMD) [3]. Feature values mainly include sample entropy, permutation entropy, energy entropy, and fractal dimension. Traditional methods for identifying and classifying fault states are mainly machine learning algorithms such as the K-nearest neighbor algorithm, support vector machine (SVM), and random forest [4]. However, these traditional fault diagnosis methods are greatly affected by noise and vibration in complex environments, and they overly rely on the work experience and professional knowledge of experts as well as knowledge of prior conditions. It is difficult to ensure efficiency and accuracy for large amounts of fault signal data, making it difficult to meet the requirements of complex modern industrial environments.

In recent years, CNNs, represented by deep learning technology, have made long-term progress and have made important contributions to the development of modern industry. Deep learning technology can automatically extract fault features from bearing signal data and automatically classify and identify fault features, removing the influence of human factors on the fault diagnosis process, liberating productivity, and reducing the professionalism of fault diagnosis, which allows non-experts to become proficient after certain training and to use trained deep learning models to complete bearing trouble diagnosis tasks. For example, Xu et al. developed a hybrid deep learning model based on a CNN and gcForest, and experimental analysis showed that the proposed hybrid deep learning model could achieve higher detection accuracy than the CNN and gcForest separately [5]. Wu et al. proposed a new multi-scale feature fusion deep residual network for rolling bearing fault diagnosis, and experimental verification showed that the diagnostic performance of the model was superior to that of several popular models [6]. Wu et al. developed a deep reinforcement transfer convolutional neural network (DRTCNN) to solve the urgent problem of completing fault diagnosis tasks using limited labeled samples [7]. Jin et al. proposed a bearing fault diagnosis method based on a synthetic minority over-sampling technique, nominal and continuous (SMOTENC), and deep transfer learning, providing an effective solution for research in the field of bearing fault diagnosis [8]. Niu et al. proposed a rolling bearing fault diagnosis method based on principal component analysis and an adaptive deep belief network with a parameter correction linear unit activation layer, and they proved the effectiveness and accuracy of the proposed method [9]. Li proposed a multi-scale attention-based transfer model (MSATM), providing a promising tool for cross-bearing fault diagnosis [10]. Tang et al. proposed a bearing fault diagnosis method based on a hybrid pooling deep belief network (MP-DBN). The results showed that the MP-DBN is a powerful rolling bearing composite fault diagnosis technology with excellent feature extraction ability and diagnostic efficiency [11]. Wen et al. proposed a new motor bearing fault diagnosis method under small samples, and the results showed the effectiveness and feasibility of the introduced method for motor bearing fault diagnosis under small samples [12].

In bearing fault diagnosis, the removal of noise interference has always been a very important issue. Noise signals are often contained in bearing signal data, seriously affecting the extraction and identification of fault signal features, greatly reducing the accuracy of fault diagnosis. For this reason, many people have made many attempts to mitigate noise interference. For example, Lei Chunli et al. proposed a rolling bearing fault diagnosis method based on a depthwise separable residual network (DS-ResNet). Experimental verification proved that the proposed method has better anti-noise performance, generalization performance, and higher diagnostic efficiency than existing methods [13]. Matania et al. proposed a novel hybrid algorithm that enables the classification of the spall type based on zero-fault-shot learning. The novel algorithm combines physics-based algorithms with machine learning to overcome the lack of faulty data [14]. Berredjem et al. proposed an Improved Range Overlaps method (IRO) to select input feature vectors by giving them validity degrees. The Similarity method partition was found to be confused by features presenting range overlap. Consequently, they proposed a new Improved Range Overlaps method, which was found quite suitable for improving the classifier accuracy [15]. Chen et al. designed a neural network model named multi-scale CNN-LSTM (convolutional neural network–long short-term memory) and a deep residual learning model. Experimental results showed that this model has better anti-noise performance and better rolling bearing fault diagnosis generalization ability [16]. Tang et al. proposed a credible multi-scale quadratic attention embedded CNN (TMQACNN) for bearing fault diagnosis. Experimental results showed that under the interference of noise superimposed on small samples or load changes, the proposed network is superior to six state-of-the-art networks [17]. Shan et al. proposed a bearing fault diagnosis method based on acoustic fingerprint features and deep learning and proposed the Mel-CNN model for application to motor noise data for bearing faults [18]. Wang et al. proposed a deep feature enhancement reinforcement learning method for rolling bearing fault diagnosis, establishing a deep Q network. Experimental results showed that the proposed method is superior to other intelligent diagnosis methods [19]. Xu et al. proposed a new model called Deep Spiking Residual Contraction Network (DSRSN) based on spiking neural networks (SNNs) for bearing fault diagnosis. Tested on fault signal datasets with different noise intensities, the proposed model achieved higher recognition accuracy [20]. Zhai et al. proposed an Adaptive Depthwise Separable Dilated Convolution and multi-grained cascade forest ADSD-gcForest fault diagnosis model, comparing the effects of three bearing fault diagnoses under various noise and load conditions. Experimental results demonstrated the effectiveness and practicality of the proposed method [21]. Zhang et al. proposed an improved noise environment bearing fault diagnosis method based on a dilated convolutional neural network, called MAB-DrNet. Experimental verification showed that it can maintain high diagnostic accuracy even in high-noise environments [22]. However, the various models detailed above can maintain accuracy at a high level in low-noise environments, but when facing strong-noise environments, such as −6 db or even −8 db, their diagnostic accuracy will significantly decrease. Also, some models overly emphasize the structure of the feature representation component to achieve the denoising task, neglecting the design of the feature extraction module, for example, resulting in deficiencies in aspects such as the required running time, diagnostic accuracy, and generalization ability.

To address these shortcomings, this research presents a new attention mechanism, CABA, combines it with ResNet to construct the ResNet-CABA network, combines this network with the attention gate recurrent unit (MAGRU) in a dual-channel manner, integrates the Gram denoising module (GMNR) into the bearing trouble diagnosis model, and fuses the information from the two channels through feature fusion to obtain data after dual-channel fusion. At the same time, the fully connected layer in the classification recognition module is replaced by a global average pooling layer to avoid overfitting. The experimental validation utilized the Case Western Reserve University (CWRU) bearing vibration dataset as the benchmark to rigorously evaluate the model’s diagnostic reliability across heterogeneous noise conditions. Through comparative analyses with state-of-the-art bearing fault detection frameworks, the proposed methodology demonstrated superior performance metrics.

## 2. Basic Theory

### 2.1. Gram Denoising Module

**Figure 1:**
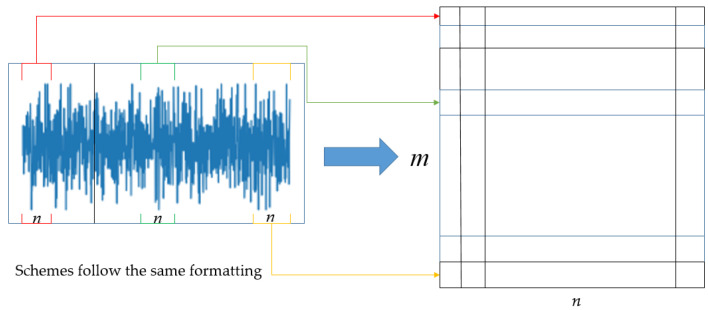
Gram matrix.

Gram denoising theory achieves denoising by adopting matrix transformation methods. The first step is to segment the 1D vibration signal of the rolling bearing according to rules and then arrange the segments according to their respective orders. This can result in the following m×n matrix. As shown in Figure 1:

(1) $$X=\left[\begin{array}{cccc} x_{1} & x_{2} & \ldots & x_{n}\\ x_{n+1} & x_{n+2} & \ldots & x_{2n}\\ \vdots & \vdots & \ddots & \vdots \\ x_{(m-1)n+1} & x_{(m-1)n+2} & \ldots & x_{mn} \end{array}\right]$$

Here, L = mn.

By enhancing the signal using the Gram matrix, the signal enhancement matrix of the input signal can be obtained as follows:

(2) $$\bar{X}=(XX^{T})X(X^{T}X)$$

When a rolling bearing fails, a broadband pulse force will occur, causing the equipment to resonate at high frequencies. When the bearing rotates, different fault types result in different frequencies of the collected periodic pulse signals. However, the vibration signal of the bearing has periodic self-similarity. Therefore, each row of the matrix *X* obtained from the periodic signal is approximately the same. According to matrix theory, similar rows or columns of the matrix make the rank of matrix *X* relatively small, and most of the energy is concentrated on a small number of large singular values of the matrix. However, after the vibration signal is polluted by noise, the rank of the signal matrix is mostly distributed among the small singular values. Therefore, as long as the proportion of larger singular values is increased and the proportion of smaller singular values is decreased, noise filtering goals are realizable. This is the principle of the Gram noise reduction theory.

For the i-th singular value $\sigma_{i}$ of *X*, the definition of the ordinary nuclear norm is

(3) $${\left\|x\right\|}_{k}=\sum_{i}\sigma_{i}$$

If the nuclear norm is small, then the rank of matrix *X* will be low. Therefore, the nuclear norm can be used to solve the low-rank problem of the matrix, thus solving the noise reduction problem in the trouble diagnosis of rolling bearings [23].

### 2.2. ResNet-CABA Network

#### 2.2.1. Residual Neural Network

Increasing the depth of the neural network architecture complicates the training process, leading to accuracy saturation and potential model degradation. The residual network, which is composed of several residual modules of network layer identity mapping, solves this problem by fast connection.

**Figure 2:**
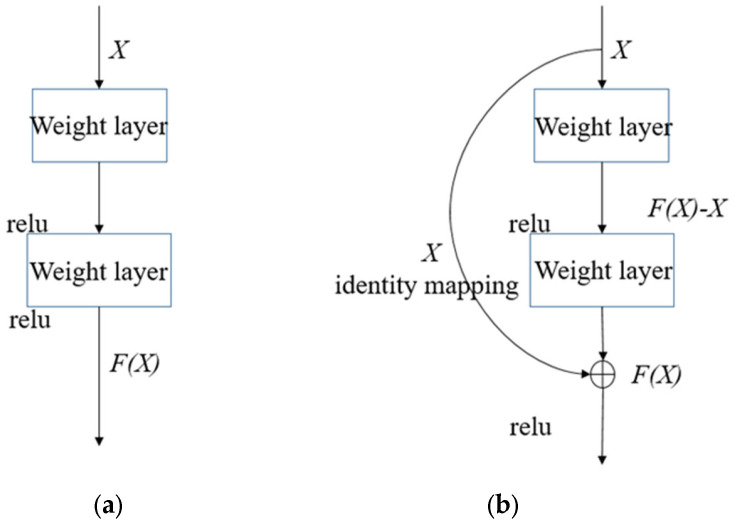
Normal block and residual block. (**a**) Normal block. (**b**) Residual block.

The residual module does not choose to fit the direct mapping of hierarchical layer architecture, but rather fits the residual mapping. As shown in Figure 2, given an initial input *X*, the target function *F(X)* denotes the theoretically perfect output mapping. In the left-hand component of the diagram, a conventional neural module directly parameterizes the target transformation *F(X)*. Conversely, the right-hand architecture is designed to learn the residual function F(X)−X through differential optimization. In reality, the residual mapping has a greater possibility of being optimized. The input in the residual block can be propagated forward at a faster speed along the cross-layer data path. This effectively reduces the difficulty of learning the mapping and speeds up the convergence of the model.

**Figure 3:**
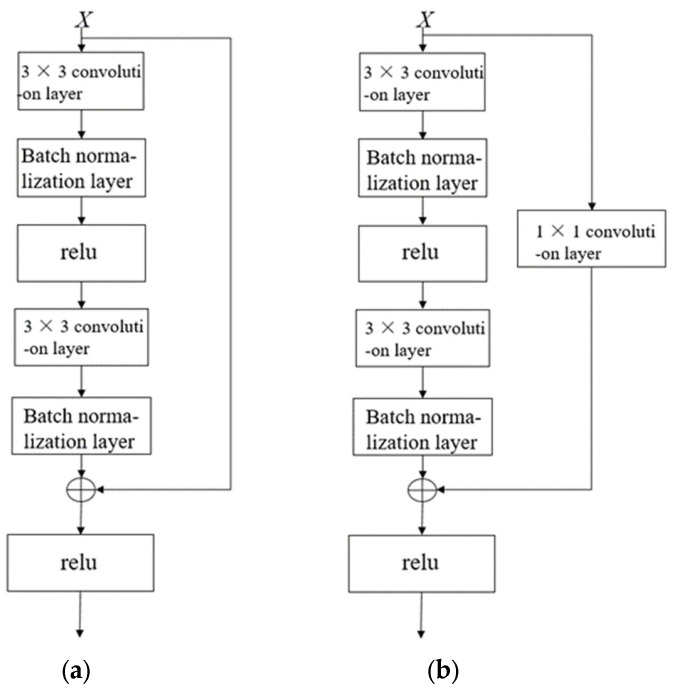
Standard residual block and residual block with downsampling layer. (**a**) Standard residual block. (**b**) Residual block with downsampling layer.

As depicted in the network topology visualization in Figure 3, the subsampling operator within residual blocks performs feature map alignment via two mechanisms: spatial resolution preservation through stride manipulation and channel matching via learnable linear transformations.

#### 2.2.2. CABA (Convolutional Avgpool Block Attention)

The attention mechanism is summarized according to the customary law of human observation of the environment. The core mechanism involves strategically amplifying the weighting of critical components to optimize the extraction of actionable insights. From a mathematical point of view, the attention mechanism operates through context-aware weighting and dynamic aggregation of input features. The main function of the attention mechanism fundamentally operates through differentiated weighting of input elements to modulate their contextual influence on different local learning of the input sequence, and then the distinct positional segments within the source sequence are multiplied by their corresponding weight values to obtain the corresponding local feature vectors, thereby amplifying and reducing the distinct positional segments within the source sequence.

**Figure 4:**
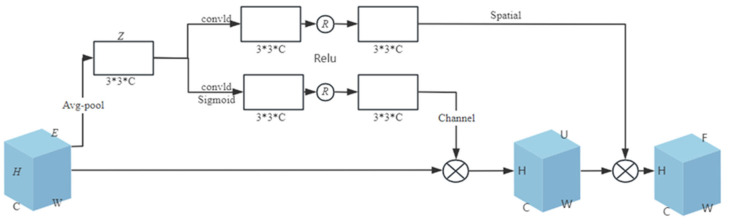
CABA schematic diagram.

Traditional attention mechanisms (such as SE attention, channel attention, etc.) have problems such as high computational complexity, dependence on local information, and poor adaptability. These problems will increase the computational cost when the model deals with the larger features of the input, and the performance is different on different datasets, which makes the model pay too much attention to some features and neglect the other important information, affecting the computational performance and stability of the model. Accordingly, this study introduces a new attention mechanism, CABA, whose principle is shown in Figure 4. Through adaptively weighting feature channels based on their contextual relevance and adaptively assigning the corresponding weight value to the feature channel, the feature channel with a larger weight value is focused on by the network; that is, task-relevant feature channels are prioritized, while irrelevant ones are suppressed. Moreover, this attention module combines channel and spatial features simultaneously, which further improves the feature representational capacity and generalization performance of the model.

An input feature *E*, which has a height of *H*, a width of *W*, and a number of feature channels of C, is given in the figure. The first step is to use global average pooling to compress the two-dimensional feature $e_{i}(i\in \left[1,C\right])$ of each channel of feature *E* and reduce the feature of the channel to a scalar $z_{i}$. The mathematical expression is as follows:

(4) $$z_{i}{=F}_{{\mathrm{avg}(e}_{i})}=\frac{1}{H\times W}=\sum_{j=1}^{H}\sum_{k=1}^{W}e_{i}$$

In the preceding equation, subscript *i* denotes the positional identifier of channels; $e_{i}$ is the global statistical information of the *i*th channel. This is a kind of spatial dimension feature compression. Since all the values of the two-dimensional feature $e_{i}$ are calculated based on the scalar $z_{i}$, it has a global receptive field to some extent. Then, the global average pooling operation is completed for feature *E* to obtain the global information *Z* of feature *E*; $Z=\left[z_{1}{,z}_{2},\ldots {,z}_{c}\right]\in R^{C}$, and $R^{C}$ is a vector space composed of *C* real numbers.

Then the channel-gated linear transformation and spatial linear transformation are performed on the global information *Z*. Usually, the linear transformation is performed through the fully connected layer, but there are problems such as high computational complexity, a lack of position information between features, and easy overfitting. To address these limitations, our methodology employs 1D convolutional layers as substitutes for fully connected layers, coupled with expanded 3 × 3 kernels. This architectural modification achieves dual objectives, namely significant parameter reduction (O(n^2^)→O(n)) and enhanced computational throughput and generalization ability of the model, and enhances the local perception ability of the model for input data.

The channel-gated linear transformation uses one-dimensional convolution to linearly transform the global information *Z* and then uses the Sigmoid activation function to nonlinearly map the export. Finally, the Relu function is employed to turn the mapping result into a likelihood value in the range of (0, 1), which is used to characterize the emphasis of every pathway, so as to obtain the weight of channel attention. This is multiplied by the entry signature *E* to generate the signature *U* containing the channel information. The formula of channel-gated linear transformation is as follows:

(5) $$f_{{(z}_{i})}{=W}_{1}z_{i}{+b}_{1}$$

(6) $$a_{i}=\frac{1}{{1+e}^{{-f(z}_{i})}}$$

(7) $$f_{{(a}_{i})}{=\mathrm{Relu}(f}_{{(a}_{i})})$$

In the above formula, $W_{1}$ and $b_{1}$ refer to the weight and bias of the convolution kernel respectively.

The spatial linear transformation, similar to the above, also uses one-dimensional convolution to perform linear transformation on the global information *Z* and then uses the Relu function to transform the transformation result into the weight of the spatial attention, which is multiplied by the signature *U* to generate the signature *F*. The signature *F* has both channel information and spatial information. The algebraic formulation governing linear spatial transformations is expressed by

(8) $$c_{i}{=g}_{{(z}_{i})}{=W}_{2}z_{i}{+b}_{2}$$

(9) $$f_{{(c}_{i})}{=\mathrm{Relu}(f}_{{(c}_{i})})$$

In the above formula, $W_{2}$ and $b_{2}$ refer to the weight and bias of the convolution kernel respectively.

### 2.3. Attention Gated Loop Unit

**Figure 5:**
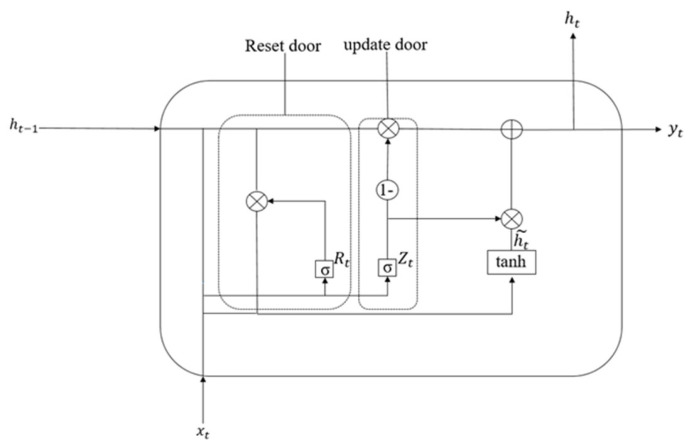
GRU schematic diagram.

The gated recurrent unit (GRU) represents a streamlined adaptation of long short-term memory (LSTM) architectures, maintaining temporal dependency modeling capabilities while eliminating redundant gating mechanisms. However, unlike the latter, the GRU merges the forgetting gate and the output gate into an update gate, integrates the unit state and output into a hidden state, and realizes the function of transmitting information by hiding information. The system of the GRU is shown in Figure 5.

The principle formula of the GRU is shown as follows:

Update the gate formula:

(10) $$Z_{t}{=\sigma (W}_{z}\times \left[h_{t-1}{,x}_{t}\right])$$

Reset door formula:

(11) $$R_{t}{=\sigma (W}_{r}\times \left[h_{t-1}{,x}_{t}\right])$$

Candidate hidden state formula:

(12) $$h_{t}{=(1-Z}_{t})\times h_{t-1}{+Z}_{t}\times {\tilde{h}}_{t}$$

Hidden state formula:

(13) $${\tilde{h}}_{t}=\tanh(W\times \left[R_{t}\times h_{t-1}{,x}_{t}\right])$$

Here, *W*, $W_{z}$ and $W_{r}$ refer to the learnable proportion, and *σ* refers to the Sigmoid activation function.

Compared with LSTM, the GRU has obvious advantages: the possibility of overfitting is reduced due to the reduction of parameters; in the face of many training data, it can significantly improve the training speed and reduce the time required for the operation; and the GRU itself is scalable, making it possible to build a larger model.

Because the characteristics of different moments can make different contributions to the next trouble diagnosis in the research of bearing trouble diagnosis, by adding an attention mechanism layer in the GRU module, the contribution of the main time characteristics to the fault diagnosis can be improved, so the attention mechanism can be used for self-regulation to obtain the best diagnostic results. The mechanism of attention-weighted summation is as follows:

(14) $$c=\sum_{i=1}^{n}a_{i}\times h_{i}$$

In the above formula, $a_{i}$ is the attention weight distribution; $h_{i}$ is the hidden layer output [24].

### 2.4. Global Average Pooling

The traditional convolutional neural network model usually uses several fully connected layers to perform feature dimension reduction. However, the full connection layer uses the full connection method to work, resulting in the phenomenon of too many parameters being generated in the trial period, which makes the calculation process of the model too complex and prone to overfitting.

The global average pooling layer (GAP) matches the categories to the attribute chart one by one and gives the actual category meaning to each channel so that each feature map can be regarded as a category confidence map relative to the category and so that the internal parameters of each feature map do not need to be optimized. The dimensionality reduction work is well completed, while the arguments of the network model can be greatly lowered, and the global feature information can be integrated, which greatly reduces the probability of overfitting. Therefore, we choose to use the global average pooling layer instead of the fully connected layer.

## 3. Fault Diagnosis Method Based on CABA-GMNR-MAGRU

**Figure 6:**
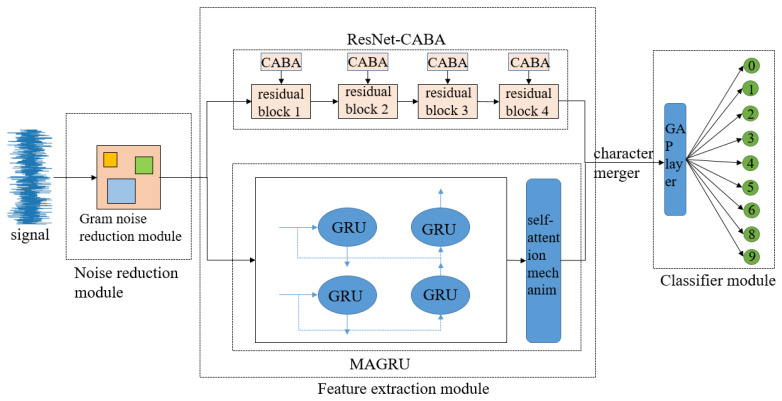
CABA-GMNR-MAGRU model structure diagram.

The CABA-GMNR-MAGRU model designed in this text is mainly composed of a denoise module, a feature extraction module, and a classifier module. The noise reduction module is composed of a Gram denoise module, and the feature extraction module is composed of a ResNet-CABA module and a MAGRU module. The classifier module is composed of a global average pooling layer and a Softmax function layer, and the precise architectural configuration is graphically delineated in Figure 6.

The vibration signature characterizing bearing degradation is ingested into the computational framework for subsequent analysis; the original signal is first denoised by the Gram noise reduction module to lower the interference of the noise signal and simultaneously optimize computational latency and predictive fidelity through architectural refinements.

The attribute abstraction module of the model is set as a dual path. One part is composed of four ResNet-CABA networks composed of four residual blocks added with the CABA module, which efficiently settles the question of network degradation, increases the computational effectiveness and generalization ability of the model, and enhances the local perception capacity of the model for incoming data. The other part is generated by two strata of gated recurrent units and a stratum of an attention mechanism layer. The gated recurrent unit has a powerful capacity to adaptively abstract temporal attributes in the data, but it has difficulty abstracting spatial attributes, and when the length of the sequential sign is greater than 200 data lengths, ‘forgetting’ may occur. Adding an attention mechanism layer can effectively settle this question and ensure the accuracy of the model’s recognition of signal data. Then the data obtained by the two pathways are fused, and the data are dimensionally spliced to obtain the fused data.

The classifier module of the model consists of two parts: the global average pooling layer and the Softmax function layer. The model inputs the data after feature fusion into the global average pooling layer for classification and then inputs it into the Softmax function layer for output using the Softmax function. Here, the global average pooling layer is selected to replace the commonly used fully connected layer. The method effectively mitigates overfitting risks and boosts diagnostic accuracy simultaneously.

Table 1 presents the detailed information of each layer of this model.

## 4. Experiment Research and Analysis

### 4.1. Experimental Environment

The environment required for the test is as follows: the programming language used is Python3.8, the deep learning framework is Tensorflow 2.10.1, the operating system is 64-bit Microsoft Windows 10, the CPU is an i5-7300H, the graphics card is a GeForce GTX1050ti graphics card, CUDA11.1 is installed on the host, and Cudnn8.2 accelerates the graphics card operation.

### 4.2. Dataset Introduction

**Figure 7:**
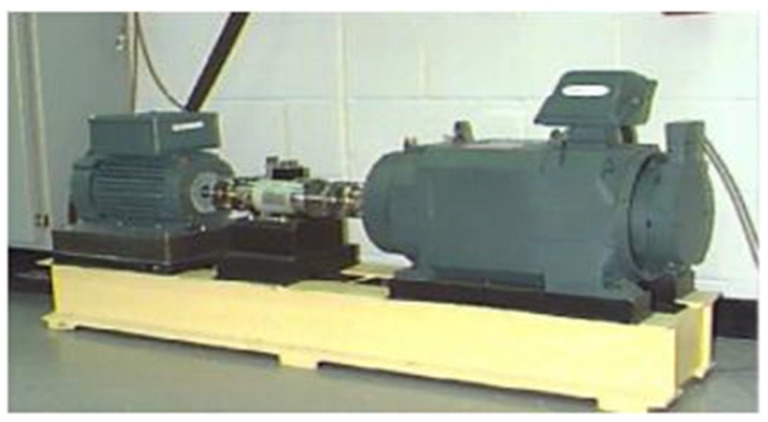
Bearing data acquisition test bench of Case Western Reserve University [25].

For this text, the rolling bearing dataset of Case Western Reserve University (CWRU) was used for experiments. The experimental platform of CWRU is shown in Figure 7. The test bench consisted of a 1.5 KW motor, torque sensor, dynamometer, and electrical control device. The bearing vibration signals of the drive end were collected by the acceleration sensor installed on the base shell, including normal, inner ring fault, outer ring fault, and rolling element fault signals. The bearing model used was SKF6205. The single-point faults of the inner ring, outer ring, and rolling body were formed by the electric spark method. The fault diameters were 0.1778 mm, 0.3556 mm, and 0.5334 mm, respectively. The sampling frequency was 12 kHz, and the load was 0 hp~3 hp. The vibration signal was collected, and each size had three faults of the inner ring, outer ring, and rolling body, for a total of nine kinds. The addition of a normal state resulted in a total of 10 sample types, as shown in Table 2.

The dataset was constructed, and the length of each type of sample was 1024, with a total of 1410 data samples. Among them, 80% of the samples were randomly selected as the training set to train the model, and 20% of the samples were used as the test set to test the model.

### 4.3. Results and Analysis of Different Models

This study conducted comparative benchmarking of the proposed deep learning architecture against established models using identical fault diagnosis datasets. This paper selected four classical deep learning models (WDCNN [26], Resnet [27], CNN-LSTM [28], BP [29]) as comparative experiments.

Firstly, under the load of 0 hp, the bearing vibration dataset under specified operational conditions underwent stratified 8:2 partitioning (training/test). And the test set was used as the training set. Comparative evaluations were conducted between our proposed DL architecture and four canonical models using identical preprocessing protocols. According to the training environment proposed above, 50 iterations were carried out, and each model was repeated for 5 experiments. The diagnosis precision and running time of every model were recorded, and data were averaged across five runs under identical test conditions. The results are shown in Table 3.

**Figure 8:**
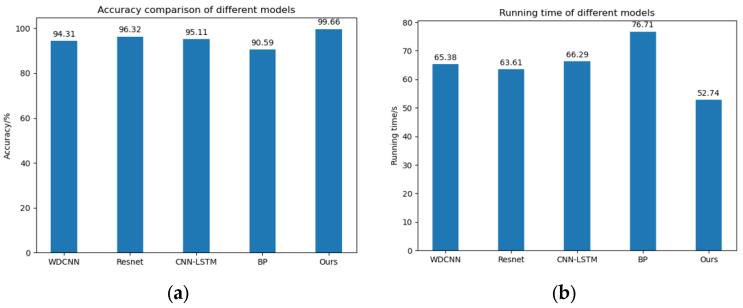
Diagnostic accuracy and running time of the five models. (**a**) Diagnostic accuracy of the five models. (**b**) Running time of five models.

The histograms of the diagnosis precision and running time of each model are shown in Figure 8.

The experimental results validate the superiority of the proposed framework; compared with the other four conventional deep learning models, it can reach an accuracy of 99.66%, while the other four models reach only 96.32%, so the proposed model has a 3% accuracy advantage. In terms of running time, this paper proposes that the CABA-GMNR-MAGRU model needs only 52.74 s to iterate 50 times, while the other four models need at least 63.61 s. The CABA-GMNR-MAGRU model has a time advantage of 10 s. It follows that the CABA-GMNR-MAGRU model proposed in this text has obvious merits over the traditional deep learning models in both diagnostic accuracy and running time. Therefore, the CABA-GMNR-MAGRU model proposed in this paper is feasible.

### 4.4. The Operation Results and Analysis of This Model Under Different Working Conditions

**Figure 9:**
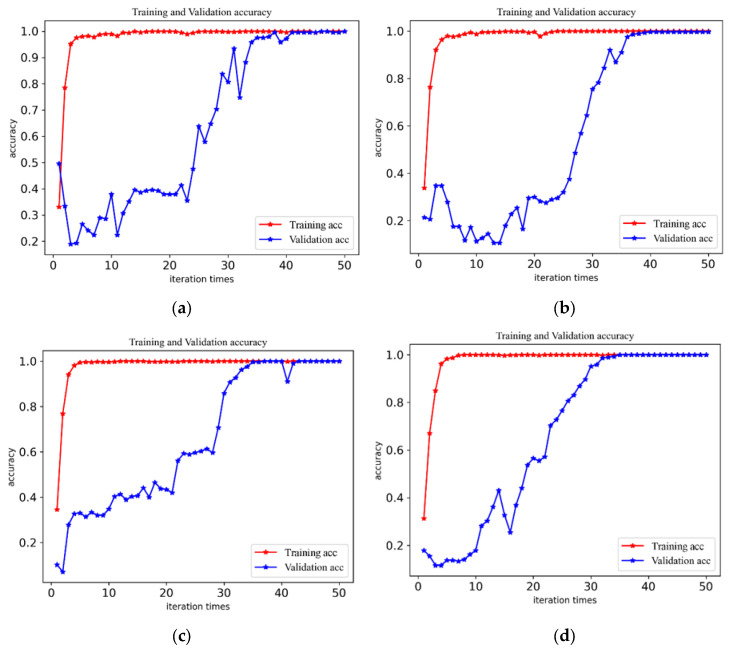
The diagnostic precision of the model under four loads. (**a**) The diagnostic precision curves of the model when the load is 0 hp. (**b**) The diagnostic precision curves of the model when the load is 1 hp. (**c**) The diagnostic precision curves of the model when the load is 2 hp. (**d**) The diagnostic precision curves of the model when the load is 3 hp.

To check the accuracy and stability of the fault diagnosis of the CABA-GMNR-MAGRU model proposed in the paper in the various working environments, vibration signal datasets with a load of 0 hp–3 hp were used to verify the model. The training environment and other configurations were the same as above. Under each load, the model proposed in this paper was used for 50 iterations, and the precision lines of the model in the four load environments were obtained, as shown in Figure 9.

**Figure 10:**
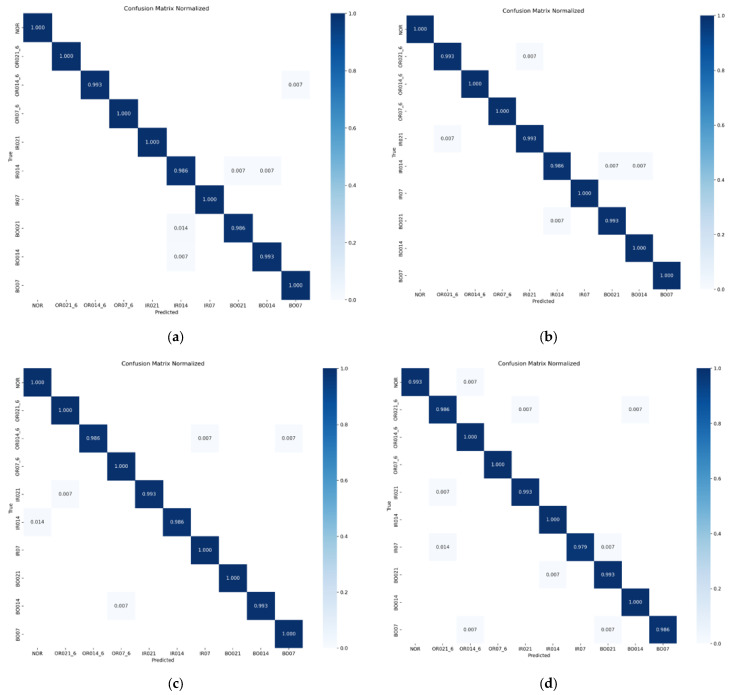
Model diagnosis confusion matrix under four loads. (**a**) The diagnostic confusion matrix of the model when the load is 0 hp. (**b**) The diagnostic confusion matrix of the model when the load is 1 hp. (**c**) The diagnostic confusion matrix of the model when the load is 2 hp. (**d**) The diagnostic confusion matrix of the model when the load is 3 hp.

The figure demonstrates that stability is achieved beyond around 35 iterations, and the accuracy rates are 99.66%, 99.66%, 99.57%, and 99.31%, respectively, all of which maintain a very high accuracy. With the increase in speed, although the diagnostic accuracy rate has a certain decline, it can still maintain a very high level. Furthermore, to show the model’s identification of bearing faults more clearly and accurately, the confusion matrix of model diagnosis under four loads is presented in Figure 10.

It can be seen from the diagram that under the four loads, the number of label recognition errors in the CABA-GMNR-MAGRU model proposed in this paper is very low. The proportion of single label recognition errors is only 2.1% of the samples under the label. On the whole, under the four loads, the proportion of model recognition errors is only 0.69% of the total samples.

Therefore, in summary, the CABA-GMNR-MAGRU model proposed in this paper can maintain a very high trouble diagnosis precision under different speed environments, and the diagnostic accuracy fluctuates little with the change of speed, which demonstrates the model’s robustness and reliability across varying operational scenarios. Hereby, the CABA-GMNR-MAGRU model proposed in this paper is feasible.

### 4.5. The Operation Results and Analysis of This Model in Noise Environment

**Figure 11:**
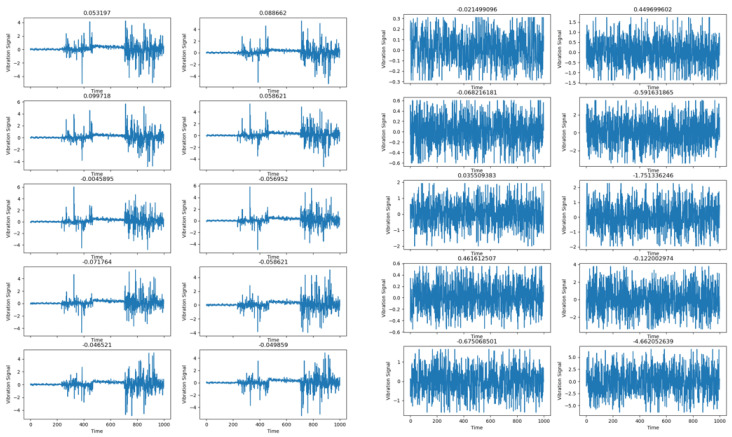
Data timing diagram of dataset before and after adding Gaussian noise.

Maintaining the precision of bearing trouble diagnosis in various-intensity noise environments is an important topic of fault diagnosis. The CABA-GMNR-MAGRU model proposed in this paper uses the Gram denoise module (GMNR) for noise reduction. In order to verify the noise reduction ability of the model, Gaussian noise and impulse noise with different SNRs were added to the bearing dataset used above to simulate the influence of noise on fault diagnosis under actual working conditions. The data timing diagram of the dataset before and after adding Gaussian noise is shown in Figure 11.

**Figure 12:**
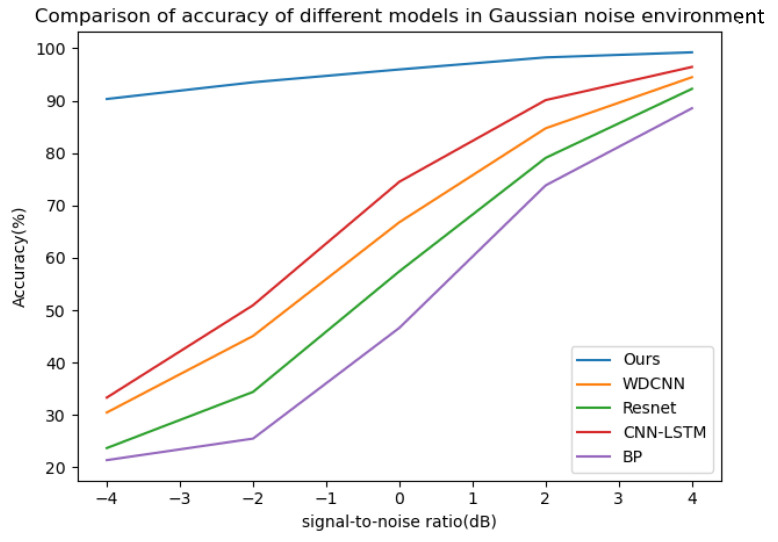
The diagnostic accuracy of the five models in different signal–noise ratios in a Gaussian noise environment.

**Figure 13:**
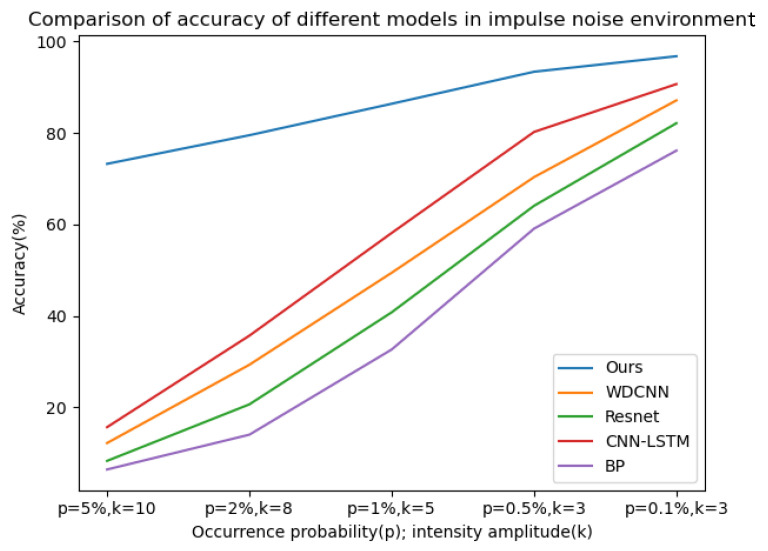
The diagnostic accuracy of the five models in different signal–noise ratios in an impluse noise environment.

Gaussian noise with SNRs of −4 db to 4 db and impulse noise with a probability of occurrence (p) and intensity amplitude (k) of 5% and 10, 2% and 8, 1% and 5, 0.5% and 3, 0.1% and 3, respectively, were added to the bearing dataset with a load of 0 hp. The probability of occurrence here refers to the probability of contamination of each data point, and the intensity amplitude refers to the multiple of the noise amplitude relative to the standard deviation of the signal. Fourteen datasets were used to verify the model, and the training environment and other configurations were the same as above. The model proposed in this paper was used for 50 iterations in each SNR and combined noise environment, and then the four classical deep learning models (WDCNN, Resnet, CNN-LSTM, BP) mentioned above were used for 50 iterations in the noise environment of the same SNR and combined as a comparative experiment; five experiments were carried out, and the average value of the experimental results was taken. The curves of diagnostic accuracy of the five models in different Gaussian noise intensity and pulse environments are shown in Figure 12 and Figure 13.

It can be seen from the above line charts that the CABA-GMNR-MAGRU model proposed in this paper can maintain a high diagnostic accuracy of about 90% under an extremely high Gaussian noise environment (−4 db), while for the other four models, the highest accuracy is only about 30%, and the lowest is even only about 21%. With the gradual weakening of noise, the diagnostic accuracy of the five models increases. The diagnostic accuracy of the CABA-GMNR-MAGRU model proposed in this paper is able to reach about 97% in a 4 db noise environment, and compared with the diagnostic accuracy in a noise-free environment, the gap is small. The remaining four models have been greatly improved compared with the diagnostic accuracy under −4 db, but there is still a big gap in their diagnostic accuracy in a noise-free environment. In the impulse noise environment with greater interference in fault diagnosis, the diagnostic accuracy of the other four models is as low as 6%, and the highest is only 15% in the extremely high impulse noise environment with *p* = 5% and *k* = 10, indicating that these models are completely unable to work normally. Even with the weakening of noise, the diagnostic accuracy of most models cannot reach more than 90%. The CABA-GMNR-MAGRU model proposed in this paper has a diagnostic accuracy of 73% even in the extremely high impulse noise environment with *p* = 5% and *k* = 10. With the decrease in noise, in the slight impulse noise environment with *p* = 0.1% and *k* = 3, its accuracy, about 96%, is also much higher than the accuracy of the other four models. Therefore, compared with other fault diagnosis models, the CABA-GMNR-MAGRU model proposed in this paper can maintain higher diagnostic accuracy in the face of a noise environment, especially in a strong-noise environment, so it has better anti-noise ability and robustness.

### 4.6. Results and Analysis of Different Model Migration Tests Under Different Working Conditions

**Figure 14:**
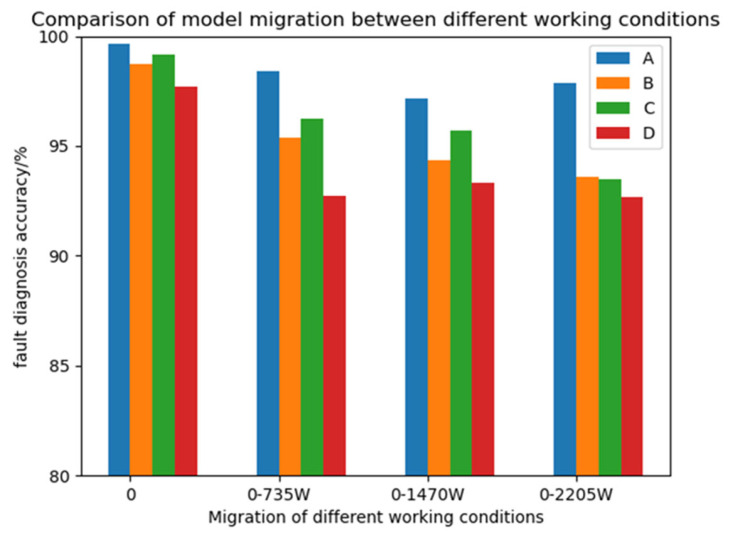
Different model migration tests in the various working environments.

Using the bearing datasets of four different loads used above, the model was trained under the condition of 0 hp load and then directly migrated to the conditions of 1 hp (735 W), 2 hp (1470 W), and 3 hp (2205 W) load. All the data were the data of the validation set, and the training environment was the same as above. At the same time, three classical attention mechanism modules, CBAM [30], SE [31], and ECA [32], were selected to replace the CABA module in the CABA-GMNR-MAGRU model proposed in this paper as a comparative experiment. For the convenience of observation, the model replaced by the CBAM module is named Model B, the model replaced by the SE module is named Model C, the model replaced by the ECA module is named Model D, and the CABA-GMNR-MAGRU model proposed in this paper is named Model A. The specific results are shown in Table 4 and Figure 14.

From the diagram above, it can be observed that the diagnostic precision of the CABA-GMNR-MAGRU model proposed in this paper is directly migrated to the other three load conditions when there is no training set. Compared with the diagnostic precision in the environment of 0 load, although the diagnostic accuracy is also decreased, the decrease is not large, and the accuracy is decreased by no more than 2%. The diagnosis precision of the three models that replace the other three attention mechanism modules directly migrating to the other three load conditions when there is no training set is more than 6% less than the diagnostic precision of the 0-load condition. The diagnostic precision of the CABA-GMNR-MAGRU model in the condition of 0 load demonstrates a clear advantage over its three counterparts that replace the other three attention mechanism modules. The proposed CABA-GMNR-MAGRU framework demonstrates superior diagnostic precision coupled with enhanced generalization capabilities.

### 4.7. Ablation Experiment

For testing the influence of the removal of every part of the CABA-GMNR-MAGRU model proposed in this paper on the capacity of the model, the original model is named Model A, the model of removing the Gram noise reduction module (GMNR) in the model is named Model B, the model of removing the CABA module in the model is named Model C, the model of removing the residual network (ResNet-CABA) with the CABA module in the model is named Model D, and the model of removing the attention gated recurrent unit (MAGRU) in the model is named Model E. The above five models were trained and tested using the noise-free bearing dataset used above and the Gaussian noise bearing dataset with an SNR of −2 db. The training environment and other configurations were the same as above, and the number of iterations was 50. The experimental outcomes are shown in Table 5.

As shown in the table, the diagnostic precision of Model B in the noise-free environment is similar to that of Model A, but the diagnostic accuracy in the noise condition with an SNR of −2 db is only 65.94%, which is reduced by more than 30%. This shows that the Gram denoise module can efficiently decrease the influence of noise on diagnostic accuracy. In comparison to the primitive Model A, the diagnostic precision of Models C, D, and E decreased to a certain extent in both noise-free and noisy environments, which fully proves the role of each part of the model in fault diagnosis.

## 5. Conclusions

The concomitant pursuit of diagnostic precision, real-time responsiveness, and cross-domain robustness poses significant challenges for rolling element bearing fault detection under high-noise industrial conditions. A bearing fault diagnostic model based on a novel deep residual learning framework enhanced an attention-based module and Gram noise reduction module is proposed. The key takeaways can be summarized as follows:

(1) The CABA-GMNR-MAGRU model proposed in this paper has high diagnostic precision, low diagnostic time, favorable anti-noise ability, and robustness. The test precision of this model is better than that of four other classical deep learning models (WDCNN, Resnet, CNN-LSTM, BP) in both a noise-free environment and −4 db to −4 db noise environments, and the running time of the proposed model in the noise-free environment is less than that of the other deep learning models. Under varying SNR conditions, this model can sustain diagnostic precision, that is, it has good robustness, while the diagnostic accuracy of other models will fluctuate greatly.

(2) The CABA-GMNR-MAGRU model proposed in this paper has good generalization performance. For the models that replace the CABA module proposed in this paper with the three classic attention mechanism modules CBAM, SE, and ECA, the accuracy of migration in a single load environment and different load conditions is lower than that of the model proposed in this paper. This shows that the new attention mechanism, CABA, proposed in this paper can efficiently improve the diagnostic precision and generalization capacity of the model.

In spite of this, the method presented in this paper still has some shortcomings. For example, this model was only checked using the bearing dataset of Case Western Reserve University. It is not known whether the model can maintain approximate diagnostic accuracy when other bearing datasets and bearing datasets collected under actual working conditions are used. These problems need to be further discussed and studied in the future.

## Figures and Tables

**Table 1:** The detailed information of each layer of this model.

Layer (Type)	Output Shape	Parameters
**input_1 (InputLayer)**	[(None, 1024, 1)]	0
**gaf_encoding (GAFEncoder)**	(None, 512, 1)	0
**conv2d (Conv2D)**	(None, 512, 16)	160
**batch_normalization(BatchNormalization)**	(None, 512, 16)	64
**max_pooling2d (MaxPooling2D)**	(None, 256, 16)	0
**conv1d_1 (Conv1D)**	(None, 256, 16)	784
**batch_normalization_1(BatchNormalization)**	(None, 256, 16)	64
**leaky_re_lu_1 (LeakyReLU)**	(None, 256, 16)	0
**CABA_1 (CABA)**	(None, 32)	288
**dropout_1 (Dropout)**	(None, 256, 16)	0
**conv1d_2 (Conv1D)**	(None, 256, 16)	784
**batch_normalization_2(BatchNormalization)**	(None, 256, 16)	64
**add (Add)**	(None, 256, 16)	0
**leaky_re_lu_2 (LeakyReLU)**	(None, 256, 16)	0
**CABA_2 (CABA)**	(None, 32)	288
**max_pooling1d_1 (MaxPooling1D)**	(None, 128, 16)	0
**conv1d_3 (Conv1D)**	(None, 128, 32)	1568
**batch_normalization_3(BatchNormalization)**	(None, 128, 32)	128
**leaky_re_lu_3 (LeakyReLU)**	(None, 128, 32)	0
**CABA_3 (CABA)**	(None, 32)	288
**dropout_2 (Dropout)**	(None, 128, 32)	0
**conv1d_4 (Conv1D)**	(None, 128, 32)	3104
**batch_normalization_4(BatchNormalization)**	(None, 128, 32)	128
**conv1d_5 (Conv1D)**	(None, 128, 32)	544
**leaky_re_lu_4 (LeakyReLU)**	(None, 256, 16)	0
**CABA_4 (CABA)**	(None, 32)	288
**max_pooling1d_2 (MaxPooling1D)**	(None, 128, 16)	0
**conv1d_6 (Conv1D)**	(None, 128, 32)	1568
**batch_normalization_5(BatchNormalization)**	(None, 128, 32)	128
**bidirectional (Bidirectional)**	(None, 256, 32)	3264
**seq_attention (SeqAttention)**	(None, 32)	0
**add_1 (Add)**	(None, 128, 32)	0
**dropout (Dropout)**	(None, 256, 32)	0
**global_average_pooling1d(GlobalAveragePooling1D)**	(None, 32)	0
**concatenate (Concatenate)**	(None, 64)	0
**dropout_3 (Dropout)**	(None, 64)	0
**dense (Dense)**	(None, 10)	650

**Table 2:** Experimental dataset situation.

Sample Types	Sample Length	Sample Size	Training Set	Testing Set	Category Marking
**normal**	1024	470	329	141	0
**outer race defect** **(21in** **)**	1024	470	329	141	1
**outer race defect** **(14in** **)**	1024	470	329	141	2
**outer race defect** **(07in** **)**	1024	470	329	141	3
**inner ring fault** **(21in** **)**	1024	470	329	141	4
**inner ring fault** **(14in** **)**	1024	470	329	141	5
**inner ring fault** **(07in** **)**	1024	470	329	141	6
**rolling element fault** **(21in** **)**	1024	470	329	141	7
**rolling element fault** **(14in** **)**	1024	470	329	141	8
**rolling element fault** **(07in** **)**	1024	470	329	141	9

**Table 3:** Diagnostic accuracy and running time of the five models.

Model	Accuracy/%	Running Time/s
**CABA-GMNR-MAGRU**	99.66	52.74
**WDCNN**	94.31	65.38
**Resnet**	96.32	63.61
**CNN-LSTM**	95.11	66.29
**BP**	90.59	76.71

**Table 4:** Migration test results of different models under different working conditions.

	0	0–735 W	0–1470 W	0–2205 W
**A**	99.66%	98.43%	97.14%	97.86%
**B**	98.73%	95.69%	94.33%	93.62%
**C**	99.15%	96.24%	95.71%	93.49%
**D**	97.69%	92.72%	93.32%	92.68%

**Table 5:** Ablation experiment results.

	A	B	C	D	E
**quietness**	99.66%	99.57%	98.71%	96.22%	95.46%
**−2 db**	92.31%	65.94%	91.83%	89.15%	86.78%

## Data Availability

The data presented in this study are available on request from the corresponding author.

## Abbreviations

The following abbreviations are used in this manuscript:

DL	Deep learning
SNR	Signal-to-noise ratio
CABA	Convolutional Avgpool Block Attention
GMNR	Gram denoising module
MAGRU	Attention gated loop unit
CWRU	Case Western Reserve University
